# Supplementary material for: Negative Intrinsic Viscosity in Graphene Nanoparticle Suspensions Induced by Hydrodynamic Slip
Source: ACS Nano. 2025 Oct 24;19(44):38350–9. doi: 10.1021/acsnano.5c10415 (PMC12613845; doi:10.1021/acsnano.5c10415)
Supplement: Supplementary file 1 [file nn5c10415_si_001.pdf]

Supporting Information

Negative intrinsic viscosity in graphene nanoparticle suspensions induced by hydrodynamic slip

Adyant Agrawal,[*,†] Catherine Kamal,[‡] Simon Gravelle,[¶] and Lorenzo Botto[§]

†*Institute for Computational Physics, University of Stuttgart, 70569 Stuttgart, Germany*

‡*Department of Mathematics, University College London, WC1H 0AY London, United Kingdom*

¶*Université Grenoble Alpes, CNRS, Laboratoire Interdisciplinaire de Physique (LIPhy), 38000 Grenoble, France*

§*Process and Energy Department, Faculty of Mechanical Engineering (3mE), Delft University of Technology, 2628 CD Delft, The Netherlands*

Contents

1 Boundary integral (BI) simulations at finite Péclet number 2

1.1 Governing equations and numerical implementation 2

1.2 Influence of Péclet number on intrinsic viscosity 3

2 Slip length in molecular dynamics (MD) simulations 3

2.1 Determination of slip length from MD simulations 3

2.2 Tuning slip length via graphene-water interactions 3

3 Hydrodynamic flow field around the particle 5

4 Hydrodynamic restoring torque for slip discs 5

5 Orientation statistics of particles in dilute and semi-dilute suspensions 7

References 9

1 Boundary integral (BI) simulations at finite Péclet number

1.1 Governing equations and numerical implementation

At finite Pe, Brownian fluctuations create an additional contribution to the intrinsic viscosity. For the two-dimensional (2D) particle considered in the BI calculations used in the manuscript, the intrinsic viscosity including Brownian fluctuations can be expressed as[1]

$$\alpha = A \langle 1 - \cos 4\varphi \rangle + B + \frac{C}{2\mathrm{Pe}} \langle \sin 2\varphi \rangle , \tag{S1}$$

where A, B and C are dimensionless coefficients and the angled brackets $\langle \ \rangle$ represent an average over the steady-state orientation distribution function $p(\varphi)$. The third term on the right-hand side of eq. (S1) is the contribution to α entirely due to Brownian fluctuations. The coefficients A and B are defined in eq. (8) of the main text. The coefficient C is expressed as

$$C = \frac{3S^b_{xz}}{\dot{\gamma}\eta A_p}, \tag{S2}$$

where S^b_{xz} corresponds to the Brownian stresslet tensor

$$S^b_{ij}(\varphi) = \frac{1}{2} \int_{\mathcal{L}} \left[f^b_i(\varphi) x_j + f^b_j(\varphi) x_i - 2\eta (u^{\mathrm{sl},b}_i n_j + u^{\mathrm{sl},b}_j n_i) \right] \mathrm{d}L. \tag{S3}$$

Here the Brownian traction $\boldsymbol{f}^b$, and the corresponding Navier slip velocity $\boldsymbol{u}^{\mathrm{sl},b}$, corresponds to traction and surface slip from a rotating particle, rotating with angular velocity $-\dot{\gamma}$ in an otherwise stationary flow field. These quantities were evaluated by solving numerically the Boundary Integral equation given in eq. (6) of the main text for $\boldsymbol{u}^\infty = 0$ and $\Omega = -\dot{\gamma}$. The numerical evaluation of this integral is described in Ref. 1.

Evaluating α also required evaluating the terms in the angled brackets in eq. (S1). This term is evaluated by solving the Fokker-Planck equation[2] for the probability distribution function $p(\varphi)$. For our two-dimensional system, the Fokker-Planck equation is

$$\dot{p} = \left[D_r p_\varphi - \Omega^h(\varphi) p \right]_\varphi , \tag{S4}$$

where $\dot{p} = \partial p / \partial t$ is the time derivative, $[\ ]_\varphi = \partial [\ ] / \partial \varphi$, and $\Omega^h(\varphi)$ is the hydrodynamic angular velocity component along the $\hat{\mathbf{y}}$ direction for a force- and torque-free particle rotating in a shear flow at orientation angle φ. The angular velocity $\Omega^h(\varphi)$ can be computed numerically by solving the integral boundary equation given by eq. (6) in the main text, as described in detail in Kamal et al.[3],[4]. Equation (S4) is solved numerically by using a spectral method[4] to find $p(\varphi)$ and hence the terms given in the angled brackets in eq. (S1).

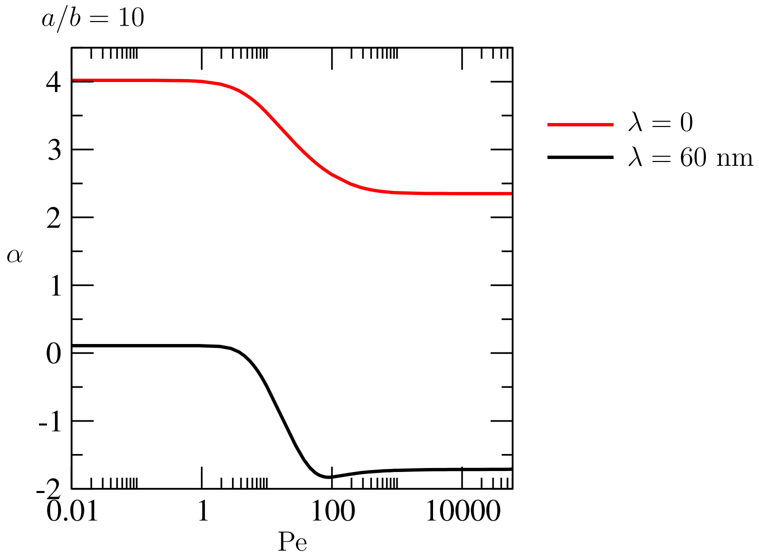

Figure S1: The intrinsic viscosity α versus Pe for $\lambda = 0$ (red curve) and $\lambda = 60$ nm (black curve) computed using the BI method with particle aspect ratio $a/b = 10$.

1.2 Influence of Péclet number on intrinsic viscosity

The method described in Eqs. S1–S4 was applied to compute the intrinsic viscosity α as a function of the Péclet number (Pe) for the model graphene particle with $a/b = 10$ and slip lengths of $\lambda = 0$ or $\lambda = 60$ nm (fig. S1). For sufficiently large Pe (i.e. for Pe$>$ 100), it was found that $\alpha(\text{Pe})$ can be well approximated by $\alpha(\text{Pe} \to \infty)$. For $\alpha(\text{Pe} \to \infty)$, eq. S1 simplifies (the term containing the coefficient C vanishes). This simplified equation is given as eq. 7 of the main text.

2 Slip length in molecular dynamics (MD) simulations

2.1 Determination of slip length from MD simulations

The hydrodynamic slip length λ at the graphene-water interface was determined using the approach described by Herrero et al.[5]. In this method, a constant body force is applied to water molecules confined between two parallel graphene walls, inducing Poiseuille flow. The velocity profile is measured in the direction parallel to the walls, and the slip length is extracted by fitting the bulk region of the profile to the analytical solution for Poiseuille flow with slip boundary conditions. To accurately define the position where the slip boundary condition applies, the Gibbs dividing surface is used as the reference. This accounts for the structured layering of water molecules near the graphene surface and eliminates ambiguities in the wall position. For unmodified OPLA-AA parameters, we obtained $\lambda = 60 \pm 11$ nm.

2.2 Tuning slip length via graphene-water interactions

The slip length λ can be tuned by adjusting the interaction parameters between graphene carbon and water oxygen atoms in the MD simulations. Specifically, the Lennard-Jones (LJ)

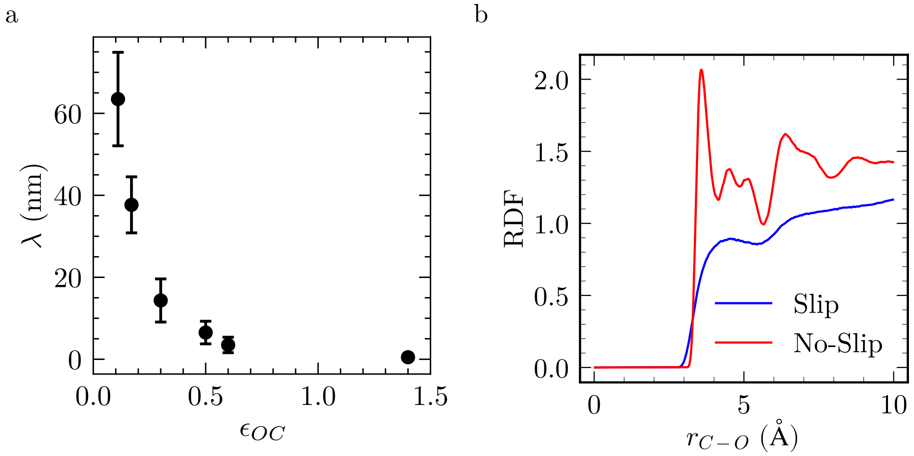

Figure S2: (a) Slip length λ, at the graphene-water interface as a function of ϵ_{CO}. (b) RDF of water oxygen atoms relative to graphene carbon atoms, comparing slip and no-slip cases.

energy parameter ϵ_{CO} was varied, which governs the strength of the interaction between the carbon and the oxygen atoms. The default LJ parameters were obtained using the Lorentz-Berthelot mixing rules applied to OPLS-AA carbon-carbon and oxygen-oxygen interactions, yielding $\epsilon_{\mathrm{CO}} = 0.114\,\mathrm{kcal/mol}$ and $\sigma_{\mathrm{CO}} = 0.335\,\mathrm{nm}$. Here, the LJ potential is defined as

$$V_{\mathrm{LJ}}(r) = 4\epsilon_{\mathrm{CO}}\left[\left(\frac{\sigma_{\mathrm{CO}}}{r}\right)^{12} - \left(\frac{\sigma_{\mathrm{CO}}}{r}\right)^{6}\right], \tag{S5}$$

where r is the distance between atoms. By increasing ϵ_{CO}, the interaction becomes stronger, resulting in reduced slip. The slip length λ was determined for various ϵ_{CO} values using the procedure described above (Fig.S2a). Increasing ϵ_{CO} leads to a monotonic decrease in λ. To impose a no-slip boundary condition, $\epsilon_{\mathrm{CO}} = 1.40\,\mathrm{kcal/mol}$ was chosen, which yields $\lambda \lesssim 0.1\,\mathrm{nm}$.

A high value of ϵ_{CO} results in strong adsorption of water molecules onto the graphene surface, effectively enforcing a no-slip boundary condition by causing the fluid to adhere to the solid interface. This effect is evident in the radial distribution function (RDF) of oxygen atoms around carbon atoms, as shown in Fig. S2b. In the no-slip case, the RDF displays a pronounced first peak with greater magnitude compared to the slip case, indicating a denser and more ordered water layer at the interface. Additional oscillations beyond the first peak reflect persistent layering of water molecules, characteristic of strong interfacial interactions. In contrast, the slip case exhibits a lower and smoother RDF, signifying weaker interactions and diminished molecular ordering at the interface. Enhanced adsorption and structuring in the no-slip scenario therefore increase the effective hydrodynamic thickness of the graphene particle.

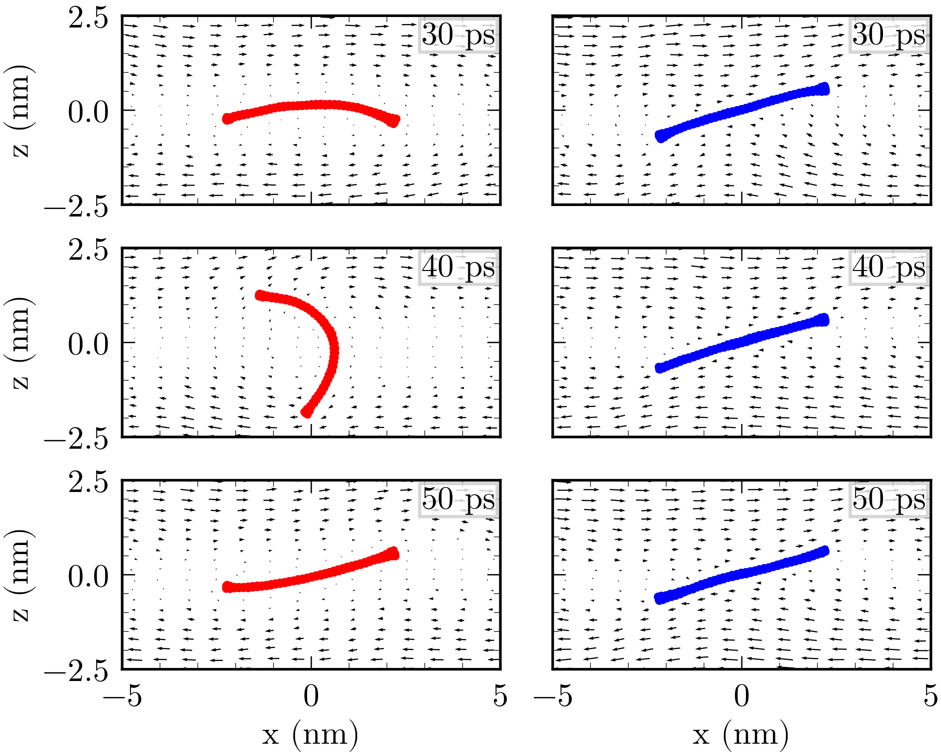

Figure S3: Fluid velocity vector field for the no-slip particle (red) and slip particle (blue) computed from molecular dynamics simulation at $\dot{\gamma} = 7 \times 10^9\,\mathrm{s}^{-1}$ and $a/b \approx 10$ at three different times.

3 Hydrodynamic flow field around the particle

Analysis of the fluid velocity fields around the particles in the MD simulations (fig. S3) reveals that hydrodynamic slip at the particle surface leads to a more uniform flow profile, thereby significantly reducing viscous dissipation in the surrounding liquid. In contrast, the periodic rotation of no-slip particles disrupts the flow and enhances viscous dissipation. A straightforward analogy can be drawn in the limit $a/b \to \infty$ and $\lambda/b \to \infty$. In this scenario, the momentum transfer between parallel fluid layers is eliminated, resulting in the viscous dissipation in the liquid approaching zero.

4 Hydrodynamic restoring torque for slip discs

To understand the stable orientation of slip discs observed in our MD simulations, we analyse the orientation dynamics of an axisymmetric slip particle in a simple shear flow using the analytical model of Kim and Karrila[6]. For our definition of particle orientation (Fig. 1c of

the main text), the polar (θ) and azimuthal (φ) satisfy the following equations of motion:

$$\dot{\varphi}(t) = -\frac{\dot{\gamma}}{k_e^2+1}\left(k_e^2 \sin^2\varphi(t) + \cos^2\varphi(t)\right) \tag{S6}$$

$$\dot{\theta}(t) = -\left(\frac{k_e^2-1}{k_e^2+1}\right)\frac{\dot{\gamma}}{4}\sin(2\theta(t))\sin(2\varphi(t)) \tag{S7}$$

Here, k_e is a parameter that can be expressed in terms of Bretherton's constant, $B = (k_e^2-1)/(k_e^2+1)$. For slip particles, k_e is purely imaginary, and θ and φ approach constant values in the limit $t \to \infty$.[4] In this limit, $\dot{\theta} \to 0$ which from eq.(S7) gives:

$$\left(\frac{k_e^2-1}{k_e^2+1}\right)\frac{\dot{\gamma}}{4}\sin(2\theta)\sin(2\varphi) = 0$$

For $|k_e| \neq 1$ and $\sin(2\varphi) \neq 0$, the steady-state solutions are $\theta = 0, \frac{\pi}{2}, \pi, \ldots$, corresponding to the particle's symmetry axis lying in the flow-gradient plane. This result explains the observed stable orientation of slip discs in our MD simulations. The component of the hydrodynamic torque driving changes in θ is given by

$$\tau_{\text{polar}} = -F_r \frac{k_e^2-1}{k_e^2+1}\frac{\dot{\gamma}}{4}\sin(2\theta)\sin(2\varphi), \tag{S8}$$

with F_r the rotational drag coefficient ($F_r \approx 32/3\eta a^3$ for thin discs of radius a [7]).

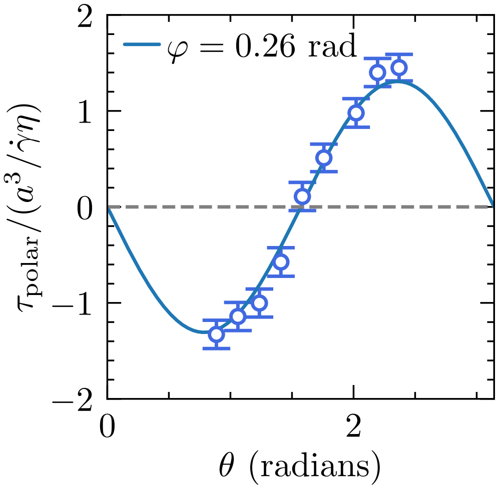

Figure S4: Normalised torque on the particle as a function of θ for $\varphi = \pi/12$ for a slip disc with aspect ratio $a/b = 10$ and at $\dot{\gamma} = 5 \times 10^10\,\mathrm{s}^{-1}$. The solid line is the theoretical prediction from eq. (S8).

Figure S4 compares τ_{polar} as a function of θ from eq. (S8) with that measured in MD for a slip disc fixed at $\varphi = \pi/12$. The MD data agrees extremely well with the analytical approximation, confirming that the observed alignment mechanism in MD is hydrodynamic in origin and captured by the continuum theory.

5 Orientation statistics of particles in dilute and semi-dilute suspensions

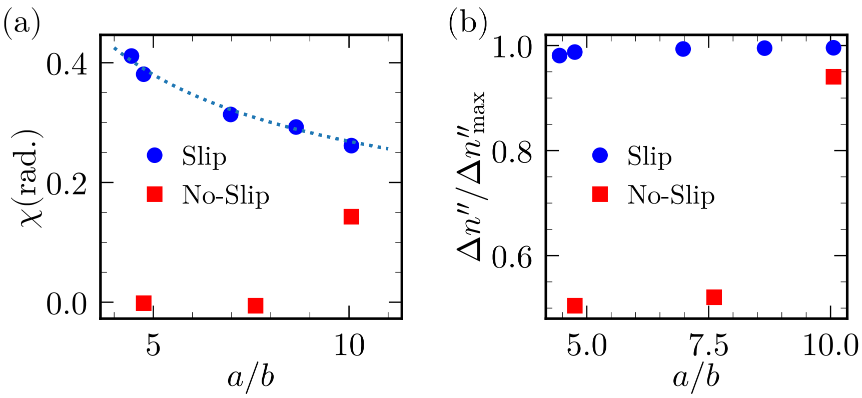

Figure S5: (a) The average orientation angle, χ and (b) the degree of alignment, $\Delta n''/\Delta n''_{\rm max}$ of slip and no-slip particle as a function of the aspect ratio a/b in MD at $\dot{\gamma} = 5 \times 10^{10}\,{\rm s}^{-1}$. The dotted blue line in (a) represents $\chi = 0.85\sqrt{b/a}$.

The orientation statistics of slip particles with different aspect ratios a/b are shown in fig. S5. In accordance with Fuller [8], the average orientation angle is expressed as

$$\chi = \frac{1}{2}\arctan\left(\frac{\langle \sin 2\varphi \rangle}{\langle \cos 2\varphi \rangle}\right), \tag{S9}$$

and the degree of alignment as

$$\frac{\Delta n''}{\Delta n''_{\rm max}} = \sqrt{\langle \sin 2\varphi \rangle^2 + \langle \cos 2\varphi \rangle^2}, \tag{S10}$$

where $\Delta n''$ is the magnitude of the linear dichorism of the suspension and $\Delta n''_{\rm max}$ denotes the value of $\Delta n''$ in a fully aligned state.

It was found that χ decreases with the increase in the aspect ratio (Fig. S5a) for slip particles. Also, the degree of alignment $\Delta n''/\Delta n''_{\rm max}$ is nearly 1 for the range of aspect ratio we explored (Fig. S5b). Therefore, the slip particles are aligned for the range of aspect ratios we explore, and the angle of alignment φ_c decreases with the increase in the aspect ratio. The continuum theory also predicts the decrease in alignment angle with the aspect ratio: At a large slip length, the effective aspect ratio is complex (See Methods section of the main paper). For $a/b \gg 1$, $|k_{\rm e}| \propto c\sqrt{b/a}$. Using (10) in the main text,

$$\varphi_c \propto \sqrt{b/a}. \tag{S11}$$

In our MD simulations, λ/a is large; we found a good agreement with eq.(S11) with the

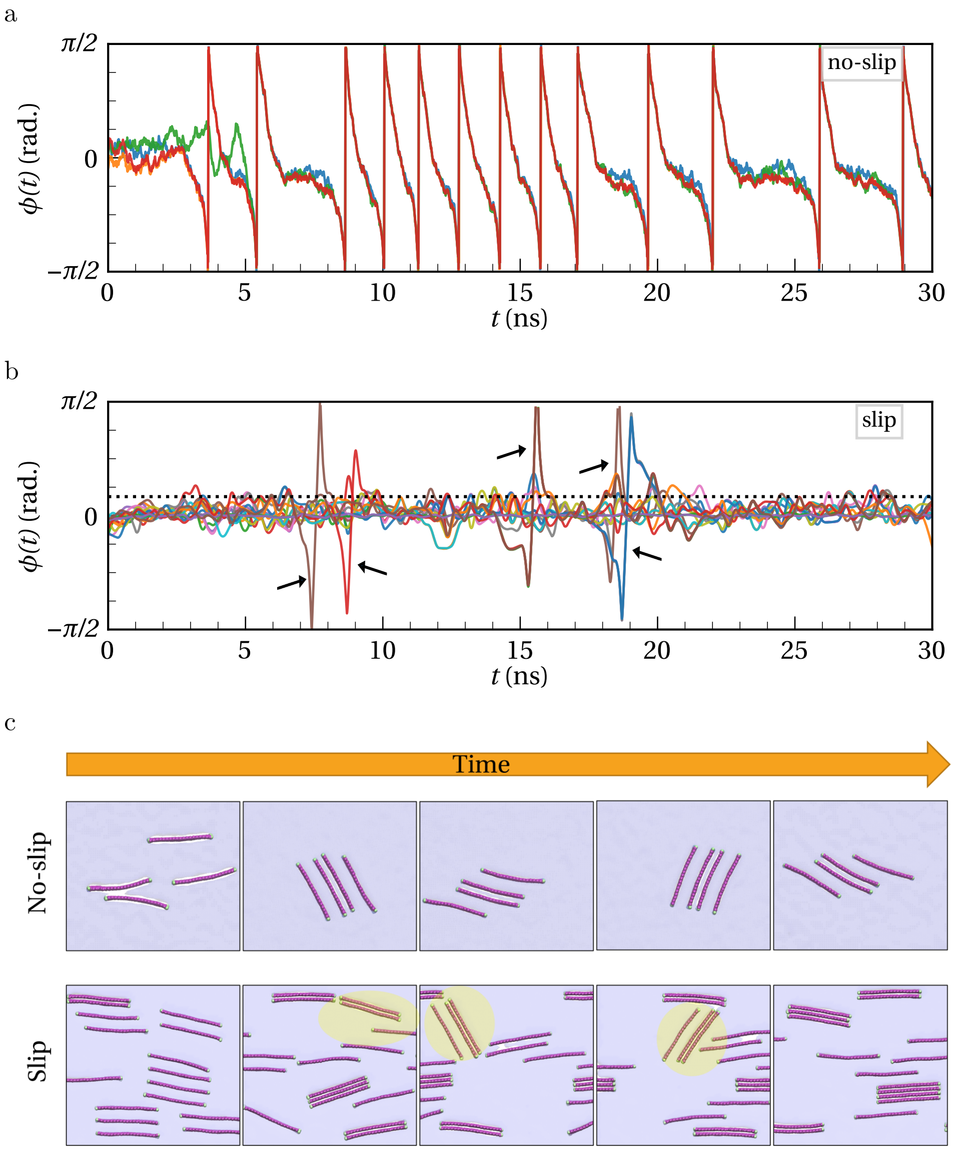

Figure S6: Time evolution of φ for each particle in quasi-2D MD simulations of (a) 4 no-slip particles at $c = 0.06$ and (b) 16 slip particles at $c = 0.24$, with $\dot{\gamma} = 7 \times 10^9\,\mathrm{s}^{-1}$. In (b), arrows mark rare full rotations, and dotted line indicates φ_c. (c) Snapshots corresponding to (a) and (b), with a full rotation event highlighted for slip case.

prefactor $c = 0.85$ (dotted blue line in Fig. S5a).

For no-slip particles, χ remains close to zero for most aspect ratios, except at $a/b = 10$, where a slight increase is observed. This deviation may be related to enhanced bending or deformation of no-slip particles within the flow-gradient plane at this specific aspect ratio. Further investigation is required to fully understand the underlying mechanisms, which we leave for future work.

The time evolution of particle orientation in semi-dilute suspensions of quasi-2D particles is shown in fig. S6 for a shear rate of $\dot{\gamma} = 7 \times 10^9\,\mathrm{s}^{-1}$. For no-slip particles, clustering occurs after a characteristic relaxation time T_R, leading to collective, periodic rotations (fig. S6a,c). In contrast, slip particles remain predominantly aligned with the shear flow direction, similar to the dilute regime (fig. S6b). The average orientation angle for slip particles is lower than the theoretical prediction of φ_c, as shown by the dotted line in fig. S6b, suggesting that the particles are aligned more in the semi-dilute regime. Nevertheless, increasing concentration enhances fluctuations in the orientation angle for slip particles, as occasional tumbling events are observed in crowded environments (indicated by arrows in fig. S6b and highlighted in fig. S6c).

References

(1) Kamal, C.; Botto, L. The effect of Navier slip on the rheology of a dilute two-dimensional suspension of plate-like particles. *J. Fluid Mech.* **2023**, *972*, A1.

(2) Gardiner, C. *Handbook of stochastic methods: for physics, chemistry & the natural sciences, (Series in synergetics, Vol. 13)*; Springer, 2004.

(3) Kamal, C.; Gravelle, S.; Botto, L. Hydrodynamic slip can align thin nanoplatelets in shear flow. *Nature Communications* **2020**, *11*, 1–10.

(4) Kamal, C.; Gravelle, S.; Botto, L. Effect of hydrodynamic slip on the rotational dynamics of a thin Brownian platelet in shear flow. *Journal of Fluid Mechanics* **2021**, *919*.

(5) Herrero, C.; Omori, T.; Yamaguchi, Y.; Joly, L. Shear force measurement of the hydrodynamic wall position in molecular dynamics. *The Journal of chemical physics* **2019**, *151*, 041103.

(6) Kim, S.; Karrila, S. J. *Microhydrodynamics: principles and selected applications*; Courier Corporation, 2013.

(7) Sherwood, J. Resistance coefficients for Stokes flow around a disk with a Navier slip condition. *Physics of Fluids* **2012**, *24*, 093103.

(8) Fuller, G. G. *Optical rheometry of complex fluids*; Oxford University Press on Demand, 1995.
